# Ameliorative Effect of Banana Lectin in TNBS-Induced Colitis in C57BL/6 Mice Relies on the Promotion of Antioxidative Mechanisms in the Colon

**DOI:** 10.3390/biom15040476

**Published:** 2025-03-25

**Authors:** Radmila Miljković, Emilija Marinković, Ivana Prodić, Ana Kovačević, Isidora Protić-Rosić, Marko Vasić, Ivana Lukić, Marija Gavrović-Jankulović, Marijana Stojanović

**Affiliations:** 1 Department of Research and Development, Institute of Virology, Vaccines and Sera “Torlak”, 11221 Belgrade, Serbia; rmiljkovic@torlak.rs (R.M.); iprodic@torlak.rs (I.P.); afilipovic@torlak.rs (A.K.); mvasic@torlak.rs (M.V.); ilukic@torlak.rs (I.L.); 2 Institute for Immunology, University Hospital Heidelberg, 69120 Heidelberg, Germany; emilija.marinkovic@uni-heidelberg.de; 3 Department of Biochemistry, Faculty of Chemistry, University of Belgrade, 11158 Belgrade, Serbia; proticrosic@chem.bg.ac.rs (I.P.-R.); mgavrov@chem.bg.ac.rs (M.G.-J.); 4 Institute of Immunology, Center for Pathophysiology, Infectiology and Immunology, Medical University of Vienna, 1090 Vienna, Austria; 5 Department of Molecular Biology, Institute for Biological Research “Siniša Stanković”—National Institute of the Republic of Serbia, University of Belgrade, 11108 Belgrade, Serbia

**Keywords:** inflammatory bowel disease, banana lectin, prophylaxis, antioxidative mechanisms, anti-inflammatory activity

## Abstract

**Background**: The global burden of inflammatory bowel diseases (IBDs), including ulcerative colitis and Crohn’s disease, is constantly rising. As IBDs significantly reduce patients’ quality of life, prevention and efficient treatment of IBDs are of paramount importance. Although the molecular mechanisms underlying IBD pathogenesis are still not completely understood, numerous studies indicate the essential role of oxidative stress in the progression of the diseases. **Objective**: The aim of this study was to investigate whether prophylactic administration of recombinant banana lectin (rBanLec) could positively affect antioxidative mechanisms in the colon and thus prevent or alleviate the severity of experimental colitis induced in C57BL/6 mice. **Methods**: The prophylactic potential of rBanLec, a mannose-binding lectin with immunomodulatory properties, was investigated in a model of 2,4,6-trinitrobenzene sulfonic acid (TNBS)-induced colitis in C57BL/6 mice. Mice received rBanLec at various doses (0.1, 1 and 10 μg/mL) before the induction of colitis. The severity of the disease was assessed by weight loss and reduction in colon length, and correlated with histopathological findings, cytokine milieu, and oxidative stress markers in the colon. **Results**: The obtained results revealed that pretreatment with a low dose of rBanLec (0.1 μg/mL) significantly reduced the severity of TNBS-induced colitis, as indicated by reduced weight loss, less severe histopathological damage, and a favorable anti-inflammatory cytokine milieu (increased IL-10 and TGFβ). In addition, rBanLec pretreatment improved the activity of antioxidant enzymes (SOD, CAT, and GST) and reduced markers of oxidative stress such as nitric oxide levels at the peak of the disease. In contrast, higher doses of rBanLec exacerbated inflammatory responses. **Conclusions**: Our findings indicate that at low doses rBanLec can alleviate the severity of colitis by modulating oxidative stress and promoting anti-inflammatory cytokine responses, positioning rBanLec as a potential candidate for treating IBDs.

## 1. Introduction

Inflammatory bowel disease (IBD) is a spectrum of chronic relapsing inflammatory diseases of the gastrointestinal (GI) tract, which primarily encompasses two major forms—Crohn’s disease and ulcerative colitis. IBDs are macroscopically characterized by rectal bleeding, severe diarrhea, body weight (BW) loss, and abdominal pain [1]. While Crohn’s disease is not associated with a specific part of the gut, ulcerative colitis is characterized by inflammation of the colonic and rectal mucosa [2,3]. The global burden of IBDs is constantly rising and represents a significant socio-economic challenge [4].

The molecular mechanisms leading to the pathogenesis of IBDs are yet to be fully explained. IBD pathogenesis is driven by complex interactions of multiple factors, including genetic background and the composition of the intestinal microbiome and exposome [5]. Evidence accumulated from animal models and clinical studies suggests that prolonged local excessive production of reactive oxygen (ROS) and nitrogen (RNS) species by infiltrated neutrophils and macrophages is a driving force in IBDs [6,7]. Moreover, macrophages and neutrophils produce proinflammatory cytokines, including tumor necrosis factor-alpha (TNFα), interleukin (IL)-1, IL-17, IL-23, IL-12, and interferon γ (IFNγ), which further promote ROS and RNS production [8,9].

Colon epithelial cells run multiple antioxidative mechanisms for the control of oxidative stress and protection of cells from ROS [6], which include the activity of antioxidative enzymes (superoxide dismutase (SOD), catalase (CAT), glutathione S-transferase (GST)), and the production of low molecular-weight antioxidant molecules (glutathione (GSH)) [10]. It is worth mentioning that the local strength of antioxidative mechanisms is highly influenced by the local cytokine milieu, in particular by regulatory anti-inflammatory cytokines such as IL-10 and transforming growth factor beta (TGFβ) [11,12,13,14]. This partially explains a positive correlation between IBD severity and excessive local production of inflammatory cytokines over anti-inflammatory cytokines [15,16].

The consumption of banana fruit is usually considered beneficial in managing GI disorders. Banana contains a spectrum of bioactive components with antioxidative and immunomodulatory properties [17,18]. Banana lectin (BanLec), a mannose-specific lectin from a jacalin-related family of lectins, is a proven immunomodulator from banana fruit [19,20,21,22,23,24]. Gavrovic-Jankulovic et al. reported the production of a recombinant BanLec (rBanLec), which structurally and functionally highly resembles its naturally occurring counterpart [20]. Both native BanLec and rBanLec are highly stable within the harsh milieu of the GI [25]. The immunostimulatory activity and immunomodulatory activity of rBanLec have been demonstrated in vitro and in vivo [23,26,27,28], with Toll-like receptor 2 (TLR2) having been identified as one of the immunologically relevant targets of rBanLec [27]. It has been shown that rBanLec, in a dose-dependent manner, triggers the production of proinflammatory cytokines, with a concomitant increase in the production of regulatory cytokines [26,27]. Its impact on the production of ROS and RNS has also been recorded in vitro in murine macrophages [27]. In addition, it has been demonstrated that rBanLec, applied in a therapeutic regime, alleviates the severity of chemically induced colitis in mice through modulation of local ROS/RNS production and promotion of the anti-inflammatory cytokine milieu [28].

Conventional treatments aim to control IBD symptoms through pharmacotherapy, including aminosalicylates, corticosteroids, immunomodulators, and biologics [29]. Conversely, a preventive strategy targeting inflammation and oxidative stress could reduce the frequency and severity of attacks. Prophylactic use of rBanLec might strengthen the colon’s antioxidant defense, potentially disrupting the cycle that drives IBD progression. With its stability in the GI tract and balanced immune modulation, rBanLec is well-suited for preventive use, especially in high-risk individuals.

Animal models of IBDs play a pivotal role in the development of new therapeutic approaches. A model of experimental colitis induced in rodents by intrarectal administration of 2,4,6-trinitrobenzene sulfonic acid (TNBS) dissolved in ethanol is a commonly used model of IBD. It mimics most IBD symptoms in humans, including histological and immunological changes [30,31]. Besides being a solvent for TNBS, ethanol disrupts the epithelial barrier and makes it leaky to intestinal antigens. Furthermore, ethanol enables haptenation of proteins within colonic tissue by TNBS, which makes them highly immunogenic. Modified proteins and intestinal antigens together promote a strong immune response in the colon, i.e., infiltration of both innate and adaptive immune cells which intensively synthesize and release a palette of soluble mediators, including (pro)inflammatory cytokines and ROS [32].

The aim of this study was to investigate whether prophylactic administration of rBanLec could positively affect antioxidative mechanisms in the colon and thus prevent or alleviate the severity of TNBS-induced experimental colitis in C57BL/6 mice. The impact of prophylactic rBanLec treatment will be evaluated through correlation of the (anti)inflammatory parameters in the colon with the severity of experimental colitis at the peak of the disease. Thus, the study provides essential data on the prophylactic potential of rBanLec with respect to IBDs, aiming to pave the way for early interventions that improve patients’ long-term outcomes and quality of life.

## 2. Material and Methods

### 2.1. Animals

Female C57BL/6 (7–8 weeks old, weighing 20 ± 2 g) mice were purchased from the Institute of Medical Research at the Military Medical Academy, Belgrade, Serbia. The experimental protocol and all procedures for animal care were approved by the Ministry of Agriculture and Environmental Protection of the Republic of Serbia (licenses No. 323-07-01577/2016-05/11 and No. 323-07-12928/2022-05/2) and performed in accordance with the 3R principles (Replace, Reduce, and Refine). Animals were in quarantine for one week prior starting of the experiment and their health status was monitored daily by trained animal care staff. They were housed at 21 °C under a 12 h light/dark cycle. Mice had ad libitum access to water and standard food, except before rBanLec treatment and induction of experimental colitis, when they were deprived of food intake overnight.

The size of the experimental groups was primarily calculated by resource equations and corrected as a contingency for eventual missing experimental animals [33]. There were no unexpected deaths or exclusions of the experimental animals during the study.

### 2.2. Production and Purification of rBanLec

rBanLec is produced in *Escherichia coli* SG13009 [pREP4] transformed with expression vector pQE70 (QIAGEN, Hilden, Germany), which contained a BanLec-encoding insert (GenBank accession number EU055641). Production and purification followed protocols described in Gavrovic-Jankulovic et al. [20]. In brief, rBanLec was produced as a 6His-tagged variant in *E. coli* SG13009. Purification involved immobilized metal affinity chromatography (elution by 0.25 M imidazole) and subsequent affinity chromatography on Sephadex G 50 (elution by 0.5 M glucose) on appropriate columns (Pharmacia, Uppsala, Sweden). Both chromatography methods were followed by dialyses against PBS. All preparations of rBanLec were evaluated using the limulus amoebocyte lysate assay (Charles River Laboratories, Wilmington, MA, USA), confirming an endotoxin contamination of less than 0.5 ng/mL, and checked for purity by electrophoresis (single band recorded at approx. 15 kDa).

### 2.3. Experimental Design

Prophylactic treatment was performed one day prior (day −1) to the induction of experimental colitis (day 0) and comprised rectal administration of rBanLec dissolved in PBS (rBanLec/PBS; 100 μL). Depending on the received rBanLec dose, mice were assigned to the rBL0.1 (0.1 μg/mL), rBL1 (1 μg/mL), and rBL10 (10 μg/mL) groups, each comprising 7 mice. Mice assigned to the positive control (PC) and negative control (NC) groups (*n* = 7 mice/group) received PBS as a prophylactic treatment. Experimental groups were formed by applying physical randomization. Mice were housed under the same ambient conditions during the experiment.

Experimental colitis was induced in mice assigned to the rBL0.1, rBL1, rBL10, and PC groups by rectal administration of TNBS (Sigma Life Sciences, Burlington, VT, USA) dissolved in 50% ethanol (25 mg/mL, 100 μL per animal), while animals assigned to the NC group received 100 μL of 50% ethanol. Mice were euthanized at the peak of the disease (day 2) [28], and colons were collected for further analysis. Collected colons were rinsed with PBS, weighed, and measured immediately upon excision. BW was recorded for each mouse before induction of experimental colitis (day 0) and euthanasia (day 2). Changes in BW were used as a primary indicator of disease severity.

Prophylactic treatment and induction of experimental colitis included rectal administrations of rBanLec- and TNBS-containing solutions, respectively, upon overnight food deprivation. Rectal administrations were conducted on mice anesthetized by intraperitoneal injection (20 μL/g of BW) of anesthesia solution (ketamine (10 mg/mL)/xylazine (1 mg/mL) in PBS). All solutions (rBanLec/PBS, TNBS/50% ethanol, PBS, and 50% ethanol) were administered slowly using a catheter carefully placed into the colon (~4 cm). Mice were held in an upside-down position for an additional 2 min to prevent leakage of the administered solution. Two independent experiments were performed (in total, 35 mice/experiment).

Mice assigned to the same group were housed in one cage. All cages were located in the same room within the animal facility in order to provide identical ambient conditions for all mice in the experiment. Periods between specific interventions and/or monitoring were comparable for all animals.

### 2.4. Histopathological Assessment of Severity of Experimental Colitis

Colons (*n* = 7 per group) were fixed in 4% formalin (24 h), dehydrated in a series of ethyl alcohol solutions (100%, 96%, and 70%; each incubation lasted for 5 min), cleared in xylene (2 × 5 min), and embedded in paraffin preheated at 55 °C. Paraffin blocks were cut into 5 μm sections using microtome Leica RM 2155 (Leica Microsystems Nussloch GmbH, Nussloch, Germany). Sections were subjected to hematoxylin/eosin (H&E) staining. The slides were examined using an Olympus BH2-RFL light microscope (Olympus Optica Ltd., Tokyo, Japan) equipped with a High-Resolution Digital Camera (Color View III, Olympus Soft Imaging Solutions, Munster, Germany). Histopathology scoring was performed by a pathologist who was blinded to the experimental groups. Calculation of the colitis histology index (CHI) was conducted in line with Koelnik et al. [34], based on the gradation of Goblet cell loss, crypt density and hyperplasia, and submucosal infiltrates (Supplement Table S1).

### 2.5. Measurement of MPO Activity in Colon Tissue

A small portion of the colon (~100 mg) was taken, weighed, and cut into small pieces. Tissue was homogenized with 0.5% hexadecyltrimethylammonium bromide in 50 mM potassium phosphate buffer, pH 6.0. Homogenates were centrifuged at 800× *g* for 30 min. Supernatants were collected and clarified by centrifugation at 13,000× *g* for 20 min. For the assessment of MPO activity, equal volumes of supernatant and 1 mg/mL OPD in 50 mM citric buffer, pH 5.0, were mixed in a 96-well microplate (Nunc MicroWell 96-Well Microplates, Thermo Fisher Scientific, Waltham, MA, USA). The reaction was initiated by adding 0.01% hydrogen peroxide and monitored spectrophotometrically at 492 nm, with reference at 620 nm. The reaction was stopped after 5 min by adding 2 M H_2_SO_4_. MPO activity was calculated by the method described by Dodda et al. [35] and expressed as the number of activity units per 1 mg of colonic tissue.

### 2.6. Homogenisation of Colonic Tissue

Homogenization of colonic tissue (*n* = 7 per group) was conducted mechanically in an ice-cold PBS solution (pH 7.4) supplemented with 1 mM of EDTA, 0.1% NP-40, 1% Triton X-100, and 1% protease inhibitors. Homogenized samples were centrifuged (18,000 rpm, 20 min, 4 °C) and supernatants were collected. The Bradford method determined the concentration of the proteins in supernatants [36]. Supernatants were stored at −80 °C until analysis. They were used for the assessment of local production ∕activity of cytokines, NO, CAT, SOD, and GST.

### 2.7. Assessment of Local NO Production

The concentration of nitrites, as an indicator of NO synthesis, was measured in the supernatants of homogenized colons (see Section 2.4) by the use of Griess reagent (1% sulfanilamide and 0.1% N-(1-naphthyl)-ethylenediamine dihydrochloride in 5% H_3_PO_4_). Supernatants were mixed with an equal volume of Griess reagent and incubated at room temperature for 10 min. Absorbance was measured at 545 nm.

Sodium nitrite in concentrations ranging from 1 to 80 µM was processed similarly. It was used as a standard for the quantification of NO in tested supernatants. NO production was expressed as the amount (micromoles) of NO per 1 mg of proteins in the supernatant obtained upon homogenization of the colon.

### 2.8. Determination of the Activity of the Antioxidative Enzymes

The activity of antioxidative enzymes (SOD, CAT, and GST) was determined in supernatants collected upon homogenization of colonic samples (see Section 2.6), following appropriate spectrophotometric methods.

SOD activity was estimated by the epinephrine method. The oxidation of epinephrine was assessed by measuring absorbance at 480 nm [37]. CAT activity was determined by measuring the rate of H_2_O_2_ decomposition at room temperature (RT) [38]. The decomposition of H_2_O_2_ was evaluated by spectrophotometry at 240 nm. GST activity was assessed by measuring the production of 1-chloro-2,4-dinitrobenzene–glutathione complex at 340 nm [39].

The activity of all enzymes was expressed as a number of unit activity per 1 mg of proteins.

### 2.9. Analyses of Cytokines

Sandwich ELISA determined the IL-1β, IL-10, IL-12, TNFα, and TGFβ concentrations. Capture and detecting antibodies for measuring mouse IL-10, IL-12, and TNFα were purchased from BioLegend (San Diego, CA, USA) and eBioscience (San Diego, CA, USA). Capture antibodies were dissolved in PBS and coated onto microtiter plates (MaxiSorp; Nunc, Roskilde, Denmark). After overnight adsorption at 4 °C, plates were blocked with 1% BSA/PBS (for 2 h at RT). The blocking and all subsequent ELISA steps were followed by washing with 0.05% Tween 20/PBS (four separate times). Samples (supernatants of homogenized colon samples and cytokine standards) were added into blocked wells and incubated for 1 h at RT. Working dilutions of samples and detecting antibodies (biotin-labeled antibodies) and ExtrAvidin-Peroxidase (Sigma, Burlington, MA, USA) were made in 1% BSA/PBS. Following incubation with a corresponding detecting antibody (1 h at RT), ExtrAvidin-peroxidase (1 h at RT) and OPD (1 mg/mL in 50 mM citric buffer) were used for visualization. After stopping the activity of peroxidase by 2 M H_2_SO_4_, absorbance was read at 492/620 nm (Multiscan Ascent, Labsystems, Thermo Fisher Scientific, Waltham, MA, USA). The production of TGFβ and IL-1β in the colon was assessed by a mouse TGFβ ELISA Ready-SET, Go! Kit (eBioscience, San Diego, CA, USA) and a Mouse IL-1β ELISA Kit (Elabscience, Houston, TX, USA), respectively. The level of specific cytokines was expressed as an amount per 1 mg of proteins in the supernatant obtained upon homogenization of the colon.

### 2.10. Statistical Analysis

Upon examination of data distribution by Shapiro–Wilk test, the statistical significance of differences among groups at the peak of the disease was assessed by a one-way ANOVA accompanied by Bonferroni’s multiple comparison test (GraphPad Prism 8, San Diego, CA, USA). A probability (*p*) value of 0.05 was taken as a significance limit for all analyses.

## 3. Results

### 3.1. Low-Dose rBanLec Prophylactic Treatment Reduces the Severity of TNBS-Induced Colitis at the Peak of the Disease

Intrarectal instillation of TNBS resulted in colitis associated with BW loss and inflammation in the colon, irrespective of the rBanLec pretreatment. NC mice remained free from all symptoms.

**Figure 1:**
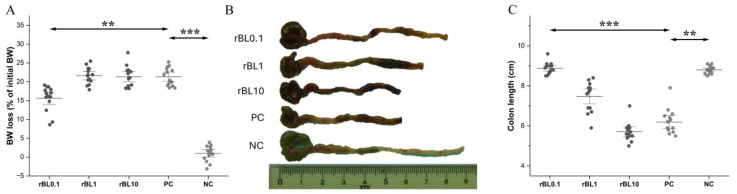
The severity of TNBS-induced colitis in rBanLec-pretreated C57BL/6 mice at the peak of the disease. Prophylactic rBanLec treatment was administered 24 h before the induction of experimental colitis (rBL0.1, rBL1, and rBL10 groups). The age-matched non-treated mice (NC) and mice subjected to TNBS administration without rBanLec pretreatment (PC) were used as controls. Body weight (BW) loss (**A**) and colon lengths (**C**) in rBanLec-pretreated mice recorded at the peak of the disease are presented as mean values ± SD (*n* = 14). Shapiro–Wilk test: BW loss—*p* > 0.05 for all groups, colon length—*p* > 0.05 for all groups. Pictures of representative colons are provided (**B**). Statistical significances of the observed differences were evaluated using one-way ANOVAs followed by Bonferroni multiple comparison tests with respect to the PC group (*p* < 0.01 **, and *p* < 0.001 ***). Two-headed arrows indicate the compared groups.

Compared to PC, prophylactic application of rBanLec in concentrations of 1 µg/mL (rBL1) and 10 µg/mL (rBL10) did not have any significant impact on BW loss at the peak of the disease (day 2). Although BW loss in rBL0.1 mice was not prevented (*p* < 0.001 compared to NC), it was significantly lower than in the PC, rBL1 and rBL10 groups (for all groups *p* < 0.001) (Figure 1A). Furthermore, the lengths of the colons taken from the rBL0.1 group at the peak of the disease were comparable to the NC colons, and significantly longer compared to the colons from the PC group (*p* < 0.001) as well as the rBL1 (*p* < 0.05) and rBL10 (*p* < 0.001) groups (Figure 1B,C). A smaller reduction in colon length was also recorded in rBL1 mice compared to the PC group (*p* < 0.05). The lengths of colons in the rBL10 mice were comparable to those recorded in the PC group and significantly reduced compared to the NC group (*p* < 0.001).

**Figure 2:**
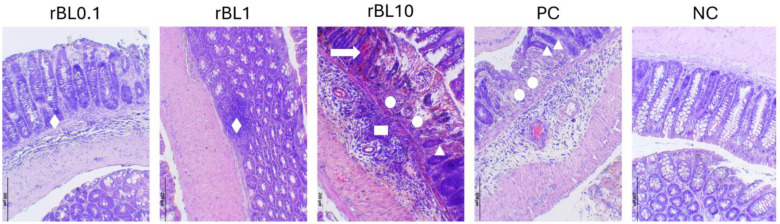
H&E-stained sections of the colons collected from rBanLec-pretreated C57BL/6 mice at the peak of TNBS-induced colitis. Prophylactic rBanLec treatment was administered 24 h before the induction of experimental colitis (rBL0.1, rBL1, and rBL10 groups). The age-matched non-treated mice (NC group) and mice subjected to TNBS administration without rBanLec pretreatment (PC group) were used as controls. Representative pictures are provided. Loss of goblet cells (△), damaged architecture of crypts (○), infiltration of polymorphonuclear cells in the area of the attenuated crypts (♢) and in submucosal layers (▭), and hemorrhaging (⇨) are indicated. Original magnification—10×; size markers indicated in lower left corner.

Histological analysis of colon sections from PC and rBL10 groups revealed structural changes, including severe inflammation with loss of goblet cells, damaged architecture of crypts, and edema in the submucosa due to the infiltration of inflammatory cells. Besides tiny ulcerations in some parts of the mucosa, the colons of rBL0.1 and rBL1 mice exhibited attenuation of the crypts with partial goblet cell loss and infiltration of polymorphonuclear cells in the area of the attenuated crypts (Figure 2). When comparing rBL0.1 and rBL1 sections, inflammatory infiltrates in the submucosal area and hypertrophy of the muscularis externa stratum were more pronounced in the rBL1 sections. Colonic sections of rBL10 mice, similar to those of PC mice, revealed abundant infiltrates of polymorphonuclear cells in submucosal layers, extreme destruction of crypts, and severe hemorrhaging in the mucosal layer. CHIs including gradation of goblet cell loss, crypt density, hyperplasia, and submucosal infiltrates [34], revealed that the destruction of colonic tissue was mildest in rBL0.1 mice (CHI = 7.0 ± 1.7), intermediate in rBL1 mice (CHI = 11.9 ± 3.0), and severe in rBL10 mice (CHI = 19.1 ± 2.3). The extent of pathological changes in rBL10 mice was comparable to that in PC mice (CHI = 20.3 ± 0.7).

The most pronounced alleviation in clinical symptoms was recorded in the rBL0.1 group. The severity of experimental colitis, estimated primarily via BW loss, was notably lower in the rBL0.1 group compared to the PC and rBL10 groups. The extent of the reduction in colon length in the rBL1 group implies that prophylactic treatment with 1 µg/mL rBanLec also exerted some beneficial impact. The results of the histological analysis were in line with clinical symptoms, showing that treatment with low doses of rBanLec resulted in decreased damage to colonic epithelia and lesser infiltration of inflammatory cells compared to the PC group. In summary, the obtained results strongly imply an inverse dose-dependent alleviation in colitis severity due to rBanLec prophylactic treatment.

### 3.2. Prophylactic Treatment with rBanLec Is Associated with Modulation of MPO Activity and NO Production in the Colon at the Peak of TNBS-Induced Colitis

**Figure 3:**
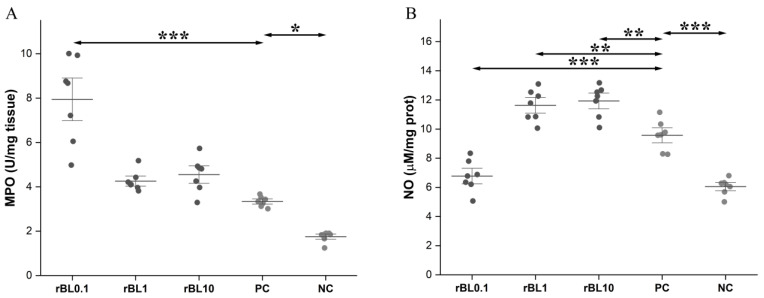
MPO activity and NO production in the colon of rBanLec-pretreated C57BL/6 mice at the peak of TNBS-induced colitis. Prophylactic rBanLec treatment was administered 24 h before the induction of experimental colitis (rBL0.1, rBL1, and rBL10 groups). The age-matched non-treated mice (NC group) and mice subjected to TNBS administration without rBanLec pretreatment (PC group) were used as controls. Local MPO activity (**A**) and NO production (**B**) are presented as mean values ± SD (*n* = 7). Shapiro–Wilk test: MPO—*p* > 0.05 for all groups except NC; NO—*p* > 0.05 for all groups. Statistical significance levels of the observed differences were evaluated using one-way ANOVAs followed by Bonferroni multiple comparison tests (*p* < 0.05 *, *p* < 0.01 **, and *p* < 0.001 ***). Two-headed arrows indicate the compared groups.

Induction of experimental colitis resulted *per se* in the rise of MPO activity in the colon at the peak of the disease (PC vs. NC *p* < 0.05). Prophylactic treatment with 1 and 10 µg/mL of rBanLec (rBL1 and rBL10 groups) did not significantly affect the local MPO activity compared to the PC group. However, treatment with 0.1 μg/mL rBanLec (rBL0.1) was associated with a significant elevation in MPO activity in the colon at the peak of the disease compared to PC mice as well as to the rBL1 and rBL10 groups (for all listed groups *p* < 0.001; Figure 3A).

Experimental colitis was also associated with a significant rise in local NO production at the peak of the disease (PC vs. NC *p* < 0.001). Furthermore, local NO production in the rBL1 and rBL10 groups also elevated, significantly exceeding the one recorded in the PC group (for both groups, *p* < 0.01; Figure 3B). However, NO production in the colon of rBL0.1 mice was significantly lower than in the rBL1, rBL10, and PC groups (for all listed groups *p* < 0.001; Figure 3B); it was comparable to the NO production recorded in the NC group.

### 3.3. Prophylactic Treatment with rBanLec Is Associated with Enhancement in the Activity of Antioxidative Enzymes in the Colon at the Peak of TNBS-Induced Colitis

**Figure 4:**
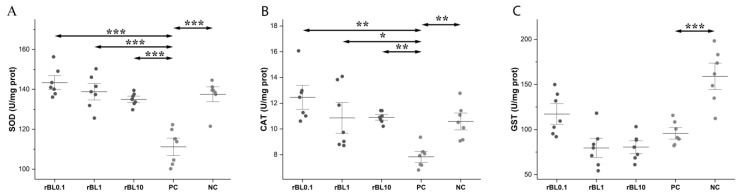
Activity of the antioxidative enzymes in the colon of rBanLec-pretreated C57BL/6 mice at the peak of TNBS-induced colitis. Prophylactic rBanLec treatment was administered 24 h before the induction of experimental colitis (rBL0.1, rBL1 and rBL10 groups). The age-matched non-treated mice (NC group) and mice subjected to TNBS administration without rBanLec pretreatment (PC group) were used as controls. Local activity of SOD (**A**), CAT (**B**), and GST (**C**) are presented as mean values ± SD (*n* = 7). Shapiro–Wilk test: SOD—*p* > 0.05 for all groups except NC; CAT—*p* > 0.05 for all groups; and GST—*p* > 0.05 for all groups. Statistical significances of the observed differences were evaluated using one-way ANOVAs followed by Bonferroni multiple comparison tests (*p* < 0.05 *, *p* < 0.01 **, and *p* < 0.001 ***). Two-headed arrows indicate the compared groups.

The activity of all measured antioxidative enzymes was significantly decreased in the colons of PC mice at the peak of the disease compared to the NC group (SOD *p* ˂ 0.001, CAT *p* ˂ 0.05, GST *p* ˂ 0.001). However, compared to PC mice, prophylactic treatment with rBanLec enhanced activity in colonic tissue (Figure 4).

Local SOD activity in all rBanLec pre-treated mice was significantly higher compared to the PC group (*p* < 0.001) at the peak of the disease, reaching the level recorded in the NC group. Among rBanLec pre-treated groups, the highest SOD activity was recorded in the rBL0.1 group, although it was not significantly higher compared to the rBL1 and rBL10 groups (Figure 4A).

In mice subjected to the rBanLec pretreatment, CAT activity was significantly higher than in PC mice (PC vs: rBL0.1 *p* < 0.01, rBL1 *p* < 0.05, rBL10 *p* < 0.01), reaching the level recorded in NC mice. Among rBanLec-treated groups, the highest CAT activity at the peak of the disease was recorded in the rBL0.1 group, although it was not significantly higher than in the rBL1 and rBL10 groups (Figure 4B).

In all rBanLec-pretreated mice, irrespective of the rBanLec dose, GST activity at the peak of the disease was significantly reduced compared to the NC group (NC vs. PC *p* < 0.001, rBL1 and rBL10 *p* < 0.001, and rBL0.1 *p* < 0.01). Compared to the PC group, rBanLec pretreatment did not significantly alter local GST activity at the peak of the disease, although it was slightly enhanced in rBL0.1 mice (Figure 4C). GST activity in the colons of rBL0.1 mice was significantly higher than the in rBL1 (*p* < 0.05) and rBL10 (*p* < 0.05) groups at the peak of the disease.

### 3.4. Prophylactic Treatment with rBanLec Shapes Characteristics of the Cytokine Milieu in the Colon at the Peak of TNBS-Induced Colitis

**Figure 5:**
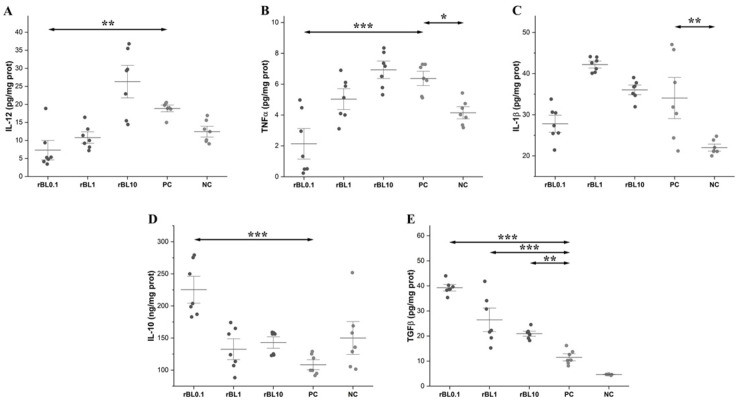
Production of the inflammatory (IL-12, TNFα, and IL-1β) and regulatory (IL-10, TGFβ) cytokines in the colon of rBanLec-pretreated C57BL/6 mice at the peak of TNBS-induced colitis. Prophylactic rBanLec treatment was administered 24 h before the induction of experimental colitis (rBL0.1, rBL1, and rBL10 groups). The age-matched non-treated mice (NC group) and mice subjected to TNBS administration without rBanLec pretreatment (PC group) were used as controls. Local levels of IL-12 (**A**), TNFα (**B**), IL-1β (**C**), IL-10 (**D**), and TGFβ (**E**) are presented as mean values ± SD (*n* = 7). Shapiro–Wilk test: IL-12—*p* > 0.05 for all groups except rBL0.1; TNFα—*p* > 0.05 for all groups; IL-1β—*p* > 0.05 for all groups; IL-10—*p* > 0.05 for all groups except rBL10; TGFβ—*p* > 0.05 for all groups except NC. Statistical significances of the observed differences were evaluated using one-way ANOVAs followed by Bonferroni multiple comparison tests (*p* < 0.05 *, *p* < 0.01 **, and *p* < 0.001 ***). Two-headed arrows indicate the compared groups.

Generally, the development of TNBS-induced colitis was associated with an increase in the local production of IL-12, TNFα, and IL-1β. Compared to NC mice, enhanced local production of inflammatory cytokines was recorded in the PC group at the peak of the disease (IL-12 *p* < 0.05, TNFα *p* < 0.05, and IL-1β *p* < 0.01). Furthermore, levels of inflammatory cytokines in the colon of rBL10 mice at the peak of the disease were comparable to the ones recorded in PC mice. The lowest levels of inflammatory cytokines were detected in the rBL0.1 group (Figure 5A–C). Local production of IL-12 was significantly higher in rBL10 mice (*p* < 0.001) and slightly enhanced in rBL1 mice compared to the rBL0.1 group. The dose-dependent impact of rBanLec pretreatment on the local production of TNFα at the peak of the disease was marked as well. Local levels of TNFα were significantly increased in the colons taken from the rBL1 (*p* < 0.01), rBL10 (*p* < 0.001), and PC groups (*p* < 0.001) compared to levels detected in the rBL0.1 group. At the peak of the disease, local levels of IL-1β in rBL1 and rBL10 mice were comparable to the PC group and significantly higher than in the NC group (for both groups *p* < 0.001). On the contrary, the level of IL-1β in rBL0.1 mice was similar to the NC group and was significantly lower than in rBL1 (*p* < 0.001) and rBL10 (*p* < 0.05) groups.

A significant rise in local production of regulatory cytokines IL-10 and TGFβ, which would reduce inflammatory reaction, was missing in the PC group at the peak of the disease (Figure 5D,E). However, it was recorded in some rBanLec-pretreated groups. The highest level of IL-10 was recorded in the rBL0.1 group, and it was significantly increased compared to the other rBanLec-treated groups (vs. rBL1 *p* < 0.001 and rBL10 *p* < 0.01) and the NC group (*p* < 0.01), as well as the PC group (*p* < 0.001). Significant enhancement of IL-10 production compared to the PC group was missing in the rBL1 and rBL10 groups. The highest TGFβ level was also found in the rBL0.1 group, and it was significantly higher than in the other rBanLec-treated groups (*p* < 0.001) and the PC group (*p* < 0.001), as well as the NC group (*p* < 0.001). It is important to emphasize that the levels of TGFβ in rBL1 and rBL10 mice also were significantly higher than the levels detected in control animals and PC mice (vs. rBL1 *p* < 0.001, rBL10 *p* < 0.01), as well as the NC (*p* < 0.001) groups (Figure 5E).

## 4. Discussion

Our results demonstrate that controlled priming of a local immune system in the colon by rBanLec could alleviate the severity of experimental colitis in C57BL/6 mice. The alleviation of clinical symptoms is associated with an enhancement of local antioxidative activity and skewing of the local milieu toward the resolution of inflammation and regeneration of colonic tissue.

Prophylactic treatment with rBanLec in a single dose ranging from 0.1 µg (rBL0.1 group) to 10 µg (rBL10 group) performed 24 h before the induction of experimental colitis did not prevent the onset of the disease, but, particularly in low doses, it reduced the severity of the disease. In a tested dose range, an inverse correlation between the applied rBanLec dose and the achieved alleviation effect at the peak of the disease was marked. Among rBanLec-pretreated groups, the mildest disease was recorded in the rBL0.1 group, while the worst outcome, similar to the one recorded for the PC group (experimental colitis without rBanLec pretreatment), was observed in the rBL10 group. The assessment of disease severity was based primarily on BW loss and shortening of the colon at the peak of the disease (day 2 upon the induction of experimental colitis). Histological analyses of the colon samples revealed that the severity of the disease positively correlated with the extent of infiltration of immune cells in the submucosal compartment.

In line with reports on the rise of MPO activity during the active phase of the disease in patients who have ulcerative colitis and Crohn’s disease, as well as in various animal models of IBDs [40,41], we recorded the rise in MPO activity in all mice subjected to the induction of experimental colitis. Furthermore, our results revealed an enhancement in local MPO activity in all rBanLec-pretreated groups compared to the PC group (experimental colitis without rBanLec treatment). Generally, MPO is an enzyme that contributes to the production of ROS/RNS, and it is considered a marker of neutrophil infiltration [42]. From that point, it was unexpected for the highest activity of MPO to be evidenced in the colon of rBL0.1 mice. Several studies in animal models have demonstrated that MPO deficiency may result in the exaggeration of inflammatory responses [43]. A beneficial anti-inflammatory effect of MPO is demonstrated in experimental lupus nephritis [44] and experimental autoimmune encephalomyelitis [45] where MPO-mediated suppression of pathogenic T cells overrides their harmful effects, as well as in LPS-, zymosan-, or non-viable *Candida albicans*-induced lung inflammation [46,47,48] and a sepsis model [49], where inflammation was more intensive in MPO-KO compared to wild-type mice. The anti-inflammatory activity of MPO in those studies correlated with various factors, including attenuated secretion of proinflammatory cytokines (IL-6, IFNγ, TNFα, and IL-1β) and chemokines (monocyte chemoattractant protein-1) and increased production of macrophage inflammatory protein 2. In addition, MPO is an enzyme that could exert intense dismutase activity depending on micro-environment characteristics as it cycles through redox intermediates that undergo a complex array of reactions [50]. In line with this, a model of MPO-deficient neutrophils revealed the accumulation of superoxide anions as they were broken down only by spontaneous dismutation, which was significantly slower than with MPO [51]. Furthermore, although the influx of neutrophils is tightly connected with the onset of inflammation, it has to be pointed out that neutrophils also exert an essential role in the resolution of inflammation [52]. The influx of neutrophils initially may contribute to the clearance of the bacteria that reach subepithelial compartments due to the compromised integrity of colonic epithelium, as neutrophils produce proinflammatory mediators that ensure the elimination of pathogens. However, neutrophils secrete a palette of mediators that allow tight regulation of their activity and contribute to attenuating the local inflammatory response, thus preventing severe tissue damage. The resolution of inflammation assumes apoptosis of neutrophils upon the elimination of pathogens and their subsequent efferocytosis, leading to macrophage polarization to an M2 phenotype (anti-inflammatory macrophages) [53,54]. In fact, neutrophils are becoming increasingly recognized for their role in the resolution of inflammation [55].

The presented results strongly imply that the beneficial impact of pretreatment with a low dose of rBanLec relies on the priming of the local immune system for the efficient induction of antioxidative machinery. That makes local tissue more resilient to potential damage due to intensive ROS production. The reduction in disease severity correlates strongly with increased activity of antioxidative enzymes, in particular SOD and CAT, which together mitigate oxidative stress at the peak of inflammation. The overall activity of antioxidative enzymes in the colon of rBanLec-pretreated mice significantly exceeded the one recorded in the PC group, with the highest occurring in the rBL0.1 group. Those results are in line with the findings showing that decreased SOD activity is associated with an increased inflammatory reaction in IBD patients [56]. Besides being responsible for efficiently removing ROS, antioxidative enzymes also negatively impact ROS production. It has been demonstrated in a model of TNBS-induced colitis that a rise in SOD activity inhibits the production of proinflammatory cytokines TNFα and IL-1β [57], which are enhancers of ROS production due to the promotion of endosomal internalization of Nox1 [58,59].

It has already been shown that rBanLec promotes the simultaneous production of both proinflammatory and anti-inflammatory cytokines in a dose-dependent manner [26]. The results presented herein imply that prophylactic application of rBanLec in a low dose supports the establishment of an anti-inflammatory cytokine milieu. At the same time, in higher concentrations, it favors a pro-oxidative inflammatory response at the peak of TNBS-induced colitis. At the peak of the disease, mice subjected to prophylactic rBanLec treatment exhibited enhanced local production of IL-10 and TGFβ. IL-10 is an anti-inflammatory cytokine with antioxidant properties [60]. Besides down-regulating production of IFNγ and TNFα, IL-10 acts as a negative regulator of the Nox1-based oxidase system in colon epithelia and suppresses IFNγ/TNFα-dependent up-regulation of ROS production [61,62]. TGFβ is usually assigned as a regulatory cytokine, although the response to TGFβ is far from simple. The biological effects of TGFβ are contextual, often leading to contradictory biological outcomes [13,14]. Considering a positive correlation between local TGFβ concentration and the extent of the alleviation of disease severity, we hypothesize that the capacity of TGFβ to promote renewal and regeneration of the intestinal tissue dominated over its ROS-dependent fibrogenic impact [13,14]. In support of our hypothesis, animal studies and examination of sections of IBD patients have shown that reduced TGFβ signaling interfered with intestinal regeneration in response to inflammation [63,64]. The production of anti-inflammatory cytokines IL-10 and TGFβ dominated in mice in the rBL0.1 group, while in the rBL10 group, production of TNFα and IL-12 (a promoter of IFNγ secretion and a marker of Th1 immune response skewing) dominated [59,65]. In line with these findings, the enhancement of local NO production, which assumes stimulation via innate receptors in an IFNγ-containing milieu, was lowest in the rBL0.1 group [66]. Those results corroborate data from the literature and strongly imply the connection between an unbalanced Th1 proinflammatory response and the development of severe pathology in the colon. Various models demonstrate that IBD severity depends on the net effect of T cells that promote disease through the secretion of inflammatory cytokines (Th1 and Th17) and T cells that downregulate inflammation by secretion of anti-inflammatory cytokines (Treg) [67,68]. Results obtained in a mouse model of spontaneous arthritis showing that TLR2 stimulation has been significant for the functioning of Tregs and the regulation of IFNγ–producing Th1 cells imply that the beneficial impact on IBDs might rely, at least partially, on the rBanLec-TLR2 interaction [69].

There have been many debates on the use of plant-based diets [70] and formulations [71] in IBD management. Although results imply their beneficial effects for IBDs, there is concern about potentially harmful effects associated with plant-derived lectins. Besides the capability to promote an anti-inflammatory milieu [72], plant lectins could promote intensive inflammatory reactions depending on their fine specificity and dosage [73]. In line with these findings and our previous results [26,27,28], the finding that low rBanLec doses reduce disease severity while higher doses worsen inflammatory reactions imply that proper dosing of lectin is critical for the treatment outcome. Furthermore, inflammation is associated with changes in the glycosylation patterns of proteins [74], which cause additional difficulties for the prediction of treatment outcomes. Despite potential concerns, the immunomodulatory capacity of lectins puts them into consideration for use in the treatment of various inflammatory diseases [75]. Current IBD treatments are mostly based on the use of immunosuppressive drugs such as corticosteroids, anti-TNF biologicals, thiopurines, and methotrexate. Strong immunosuppression puts IBD patients at high risk of cancer and infections [76]. In addition, IBD patients respond inadequately to vaccinations due to prolonged immunosuppression, which makes them susceptible to vaccine-preventable diseases [77]. IBD patients subjected to systemic corticosteroid therapy exhibit an increased risk of venous thromboembolism [78]. Serious adverse effects of currently used therapeutics imply that the development of new approaches in IBD management is crucial.

The presented results open new research directions regarding rBanLec and its antioxidant and anti-inflammatory properties for IBDs. As genetic background could influence IBD course [30], as well as the outcomes of rBanLec treatment [23,27,28], the assessment of rBanLec’s capacity to modulate the course of experimental colitis in mice other than C57BL/6 would contribute to the development of safe and efficient treatment protocols for IBDs using rBanLec. Although our data, in association with data from the literature, strongly imply a connection between cytokine milieu and the outcomes of specific rBanLec treatments, providing direct evidence on the role of particular cytokines in shaping of IBD course would significantly contribute to the understanding of the mechanisms underlying beneficial or negative impacts of rBanLec on IBDs. Furthermore, given that rBanLec promotes antioxidant enzyme activity, it would be revealing to investigate whether it directly activates the Nrf2 pathway, a critical regulator of cellular antioxidant responses. Examining the potential of rBanLec to activate Nrf2 or related transcription factors could provide insight into its role in modulating the expression of genes encoding antioxidant enzymes such as SOD and CAT. Those mechanistic insights may also position rBanLec as a modulator of redox-sensitive pathways in chronic inflammatory diseases beyond IBDs.

## 5. Conclusions

The impact of prophylactic administration of rBanLec was analysed in a model of acute colitis that mimics most IBD symptoms in humans, including histological and immunological changes [30]. Based on the observations provided, it can be concluded that prophylactic treatment with rBanLec in low doses mitigated the morphological and immunological features of TNBS-induced colitis in C57BL/6 mice. The beneficial impact of low-dose rBanLec pretreatment comprised fostering local anti-inflammatory and antioxidative mechanisms at the peak of the disease, shown by the alleviation of disease severity. The extent of disease alleviation inversely correlated to local NO production and production of inflammatory cytokines (TNFα, IL-12, and IL-1β), while it positively correlated with the activity of antioxidative enzymes (CAT, SOD, and GST) and the production of anti-inflammatory cytokines (TGFβ and IL-10) in the colon, providing further evidence for the protective role of rBanLec in the oxidative environment of colitis. While low-dose rBanLec pretreatment helped rebalance pro- and anti-inflammatory responses at the peak of the disease, higher doses of pretreatment led to impairment of this delicate balance, potentially tipping the scale towards increased inflammation and tissue damage. These findings suggest that low-dose rBanLec could serve as a potential prophylactic agent in managing IBDs by reinforcing the colon’s antioxidative defenses and controlling oxidative stress, thus providing a foundation for its use in chronic inflammation and oxidative damage conditions.

## Data Availability

Data described in the manuscript will be made available upon request to the corresponding author, M.S.

## Supplementary Materials

The following supporting information can be downloaded at: https://www.mdpi.com/article/10.3390/biom15040476/s1, Table S1: Histology scoring for estimation of disease severity.

## Abbreviations

BW	body weight
CAT	catalase
CHI	colitis histology index
GI	gastrointestinal tract
GSH	glutathione
GST	glutathione S-transferase
IBD	inflammatory bowel diseases
IFNγ	interferon γ
IL	interleukin
rBanLec	recombinant banana lectin
RNS	reactive nitrogen species
ROS	reactive oxygen species
RT	room temperature
SOD	superoxide dismutase
TGFβ	transforming growth factor beta
TLR2	Toll-like receptor 2
TNBS	2,4,6-trinitrobenzene sulfonic acid
