# Supplementary material for: Ameliorative Effect of Banana Lectin in TNBS-Induced Colitis in C57BL/6 Mice Relies on the Promotion of Antioxidative Mechanisms in the Colon
Source: Biomolecules. 2025 Mar 25;15(4):476. doi: 10.3390/biom15040476 (PMC12024995; doi:10.3390/biom15040476)
Supplement: Supplementary file 1 [file biomolecules-15-00476-s001.zip › biomolecules-3452144-supplementary.pdf]

Supplementary Table S1. Histology scoring for estimation of disease severity

	Feature graded				CHI
	**Goblet cell loss**	**Crypt density***	**Crypt hyperplasia***	**Submucosal infiltrates****	
**rBL0.1**	1.9 ± 0.4	0.9 ± 0.4	0	1.1 ± 0.4	7.0 ± 1.7
**rBL1**	2.3 ± 0.5	1.4 ± 1.5	0.1 ± 0.4	2.1 ± 0.4	11.9 ± 3.0
**rBL10**	2.9 ± 0.4	3	0.9 ± 0.4	2.9 ± 0.4	19.4 ± 2.3
**PC**	3	3	1.1 ± 0.4	3	20.2 ± 0.7
**NC**	0	0	0	0.3 ± 0.5	0.9 ± 1.4

CHI – colitis histology score; for CHI calculation * multiply by 2, ** multiply by 3.
